# Welcome note International Foundation of Integrated Care

**DOI:** 10.5334/ijic.1040

**Published:** 2012-09-04

**Authors:** Nick Goodwin

**Affiliations:** The King’s Fund, Interim Chair and Treasurer, IFIC Editor-in-Chief, IJIC

Welcome to INIC 12 in the Republic of San Marino!

I first would like to thank Paolo Pasini and his colleagues for their generous hospitality and work as hosts of INIC 12 here in the Republic of San Marino—it is sincerely and greatly appreciated.

In most countries in the world the effective integration of health and social care has remained either an unfulfilled dream or an unrealistic ambition. This conference, that brings together leading-edge case examples of how this has been achieved, will undoubtedly show that local leadership, vision and commitment can overcome the many obstacles to integrated care. It is apt that the Republic of San Marino should host us since we will have much to learn from their approach and philosophy to care than overcomes traditional boundaries and places the patient and users health and well-being at its heart.

Talking of ambition, in October 2011 the ***International Foundation for Integrated Care (IFIC)*** was instituted. Its mission statement is as follows:

The International Foundation for Integrated Care is a network that crosses organisational and professional boundaries to bring people together to advance the science, knowledge and adoption of integrated care policy and practice. The Foundation seeks to achieve this through the development and exchange of ideas among academics, researchers, managers, clinicians, policy-makers and users and carers of services throughout the World.

Over the next year, this new Foundation—led by a Founding Board from across 12 countries—will develop a range of activities in support of its mission. This will include taking over responsibility from the Julius Centre, University Medical Centre (UMC) Utrecht for the funding of the scientific journal the *International Journal of Integrated Care (IJIC)* as well as for the initiation and delivery of INIC’s Annual Congresses.

I would like to take this opportunity to personally thank UMC Utrecht for its 12-year sponsorship and support of these activities, and also give a special word of thanks to Prof. Guus Schrijvers—without whom neither the journal or network would have been born—and his colleagues Clarine Sies and Inge Kuurman for their personal commitment as conference organisers.

As the Foundation moves forward, it will also seek new opportunities including: a new website; a bi-annual World Congress (the first to be hosted in Singapore in 2013); the development of educational programmes; the creation of a knowledge-centre or observatory to support the Foundation as an expert international resource on integrated care; and to partner with other organisations in undertaking leading-edge research.

This is an ambitious vision, but one which I hope you might share and wish to get involved with!

I wish all of you an enjoyable and successful conference.

